# Further evidence that mutations in *INS *can be a rare cause of Maturity-Onset Diabetes of the Young (MODY)

**DOI:** 10.1186/1471-2350-11-42

**Published:** 2010-03-12

**Authors:** Trine W Boesgaard, Stepanka Pruhova, Ehm A Andersson, Ondrej Cinek, Barbora Obermannova, Jeannet Lauenborg, Peter Damm, Regine Bergholdt, Flemming Pociot, Charlotta Pisinger, Fabrizio Barbetti, Jan Lebl, Oluf Pedersen, Torben Hansen

**Affiliations:** 1 Steno Diabetes Centre and Hagedorn Research Institute, Gentofte, Denmark; 2 Department of Pediatrics, Second Faculty of Medicine, Charles University, Prague, Czech Republic; 3 Center for Pregnant Women with Diabetes, Department of Obstetrics, Copenhagen University Hospital, Rigshospitalet, Denmark; 4 Faculty of Health Science, University of Copenhagen, Copenhagen, Denmark; 5 Research Centre for Prevention and Health, Glostrup University Hospital, Glostrup, Denmark; 6 Endocrinology and Diabetes Unit, Department of Paediatric Medicine, Bambino Gesù Children's Hospital IRCCS, Piazza S. Onofrio 4, Rome, Italy; 7 Institute of Biomedical Science, Faculty of Health Sciences, University of Copenhagen, Copenhagen, Denmark; 8 Faculty of Health Sciences, University of Aarhus, Aarhus, Denmark; 9 Faculty of Health Sciences, University of Southern Denmark, Denmark

## Abstract

**Background:**

Insulin gene (*INS*) mutations have recently been described as a common cause of permanent neonatal diabetes (PNDM) and a rare cause of diabetes diagnosed in childhood or adulthood.

**Methods:**

*INS *was sequenced in 116 maturity-onset diabetes of the young (MODYX) patients (*n *= 48 Danish and *n *= 68 Czech), 83 patients with gestational diabetes mellitus (GDM), 34 type 1 diabetic patients screened negative for glutamic acid decarboxylase (GAD), and 96 glucose tolerant individuals. The control group was randomly selected from the population-based sampled Inter99 study.

**Results:**

One novel heterozygous mutation c.17G>A, R6H, was identified in the pre-proinsulin gene (*INS*) in a Danish MODYX family. The proband was diagnosed at 20 years of age with mild diabetes and treated with diet and oral hypoglycaemic agent. Two other family members who carried the *INS *R6H were diagnosed with diabetes when 51 years old and with GDM when 27 years old, respectively. A fourth mutation carrier had normal glucose tolerance when 20 years old. Two carriers of *INS *R6H were also examined twice with an oral glucose tolerance test (OGTT) with 5 years interval. They both had a ~30% reduction in beta-cell function measured as insulinogenic index. In a Czech MODYX family a previously described R46Q mutation was found. The proband was diagnosed at 13 years of age and had been treated with insulin since onset of diabetes. Her mother and grandmother were diagnosed at 14 and 35 years of age, respectively, and were treated with oral hypoglycaemic agents and/or insulin.

**Conclusion:**

Mutations in *INS *can be a rare cause of MODY and we conclude that screening for mutations in *INS *should be recommended in MODYX patients.

## Background

Insulin gene (*INS*) mutations have recently been described as a common cause of permanent neonatal diabetes (PNDM) and a rare cause of diabetes diagnosed in childhood or adulthood [1-4]. Heterozygous mutations in *INS *account for 15 - 20% of cases of PNDM [4,5]. Gene discovery can lead to recognition of novel phenotypes [6] and recognition of novel clinical subgroups. For example, MODY was initially clinically defined as autosomal dominantly inherited, non insulin dependent, early-onset diabetes, but now there are at least eight distinct genetic subgroups of MODY, most of which have a discrete phenotype and specialized treatment needs [6]. An R46Q *INS *mutation was recently described in a Norwegian study of 62 probands fulfilling conventional MODY criteria. In addition, they examined 223 patients from the population-based Norwegian Childhood Diabetes Registry and found an R55C *INS *mutation. One hundred blood donors were screened negative for these mutations [7]. The Italian study group on early onset diabetes has detected two *INS *mutations (A23S and G23S) in children negative for 5 type 1 diabetes (T1D) autoantibodies [1]. In addition, an R6C mutation was identified in an English MODY family and a L68M mutation was described in a family of Turkish origin with young-onset type 2 diabetes [3]. Furthermore, a recent screening for *INS *mutations in 252 patients diagnosed clinically with T1D between 6 months and 17 years of age identified 2 de novo heterozygous mutations G32S and R89C among the 25 (8%) antibody-negative patients [8]. To our knowledge, no studies have screened women with gestational diabetes mellitus (GDM) for *INS *mutation. GDM was defined as an abnormal glucose tolerance diagnosed for the first time in pregnancy.

We aimed to evaluate the prevalence and the disease-associated phenotype of *INS *mutations among diabetic patients diagnosed with MODY, anti-body negative T1D or GDM.

## Methods

*INS *was sequenced in 116 unrelated MODYX probands: 48 Danish, age at diagnosis (mean ± SD) 24 ± 19 years, BMI 24.8 ± 5.5 kg/m^2^, and 68 Czech, age at diagnosis 18 ± 8 years, BMI 22.6 ± 5.0 kg/m^2^, 83 Danish diabetic patients previously diagnosed with GDM, age at examination 40 ± 7 years, BMI at examination 28.1 ± 6.5 kg/m^2^, 34 GAD autoantibody-negative T1D patients, age at diagnosis 20 ± 16 years and 96 glucose tolerant control individuals, age at examination 46 ± 7 years, BMI 26.4 ± 4.4 kg/m^2^. All MODYX probands were from families fulfilling the conventional criteria of MODY [5] defined by: diabetes diagnosed before 25 years of age in at least one of the family members. No treatment with insulin and/or measurable C-peptide at least one year after diagnosis. Autosomal dominant inherited diabetes with known diabetes in at least two consecutive generations.

All MODY probands were screened negative for mutations in the MODY genes *HNF4A*, *GCK *and *HNF1A*. Women with GDM and all GAD autoantibody-negative T1D patients had a positive family history of diabetes including a diabetic parents and or child with diabetes. The control group was randomly selected from the population-based study Inter99 [9]. Additional family members of the probands with an *INS *mutation were examined and screened for the family-specific *INS *mutation. The participants and examined family members gave their informed written consent, and the study protocols were approved by the ethical committees. The genomic sequence of *INS *was analysed in two segments covering the two exons, exon-intron boundaries and UTRs. Sequencing analyses were performed as described [4]. Analyses of the *INS *sequence revealed four low-frequency (minor allele frequency = 0.05) and three frequent (minor allele frequency>0.05) variants (Table 1).

**Table 1:** *INS *gene variants identified in MODY (*n *= 116), T1DM (*n *= 34), GDM (*n *= 83) and controls (*n *= 96)

					MAF (%)		
**rs number**	**SNP**	**Position on chr. 11**	**Part of gene**	**MODY** **(*n *= 116)**	**T1D ab^-^** **(*n *= 34)**	**GDM** **(*n *= 83)**	**Controls** **(*n *= 92)**

Novel	c.-218A>C	2.138.995	5'UTR	0.4	0	0	0

rs689	c.-23A>T	2.138.800	Intron	27	10	33	28

rs5505	c.-9C>T	2.138.786	5'UTR	0.9	0	0.6	0^A^

Novel	c.17G>A, p.R6H	2.138.761	Exon	0.4	0	0	0

Novel	c.137 G>A, p.R46Q	2.138.641	Exon	0.4	0	0	0

rs3842752	c.*9C>T	2.137.649	3'UTR	20	7	27	21

rs3842753	c.*22C>A	2.137.636	3'UTR	26	10	34	28

SNP locations (-strand) are displayed counting from the first translated nucleotide in pre-proinsulin (*INS*). Base-pair positions are displayed counting from the p-arm telomere of chromosome 11 (according to the Base position feature in the Human (*Homo sapiens*) Genome Browser Gateway Human Mar. 2006 [hg18] assembly (http://genome.ucsc.edu/cgi-bin/hgGateway, assessed 19 June 2009). Minor allele frequencies (MAF) are given as a percentage.

^A) ^Subsequent genotyping of 198 population-based individuals revealed a MAF of 0.7%

Control individuals were collected as a subset of the population based study Inter99 of middle-aged Danish individuals.

## Results and Discussion

**Figure 1:**
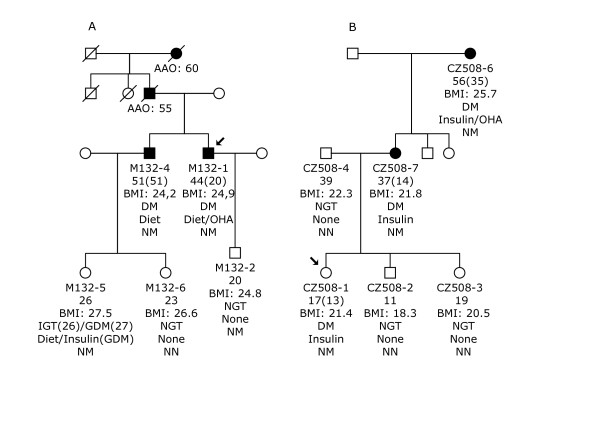
**Segregation of *INS *R6H (Figure 1A) and R46Q (Figure 1B) mutations in two MODY families**. Symbols denote the following: square, male; circle, female; empty symbol, normal glucose tolerant subject; filled symbol, diabetic person; quarter filled, impaired glucose tolerance status/gestational diabetes; symbol with arrowhead, proband. The text, under each individual represents the following: Subject id; age at examination (age at diagnosis); BMI (kg/m^2^); NGT, normal glucose tolerance; IGT, impaired glucose tolerance; GDM, gestational diabetes mellitus; DM, diabetes mellitus; treatment and mutation status; AAO, age of onset.

One novel mutation c.17G>A, R6H, was identified in *INS *in a Danish MODYX family (Figure 1A). The mutation is located in the signal peptide of proinsulin and was not present in 96 control individuals. The proband, M132-1, had been diagnosed at 20 years of age with mild diabetes treated with diet and oral hypoglycaemic agent (OHA). His 20-years-old son, M132-2, carried the mutation but had normal glucose tolerance. The brother of the proband, M132-4, carried the mutation and had been diagnosed with diabetes at 51 years of age. A daughter, M132-5, of the brother carried the mutation and had been diagnosed with IGT at the age of 26 and with GDM at the age of 27 years. Two carriers of *INS *R6H, M132-2 and M132-4, were also examined with an OGTT at age 15 years and 46 years, respectively. Interestingly, they both had a ~30% reduction in beta-cell function over a period of 5 years measured as insulinogenic index calculated from OGTT data (s-insulin 30 min (pmol/l) - s-insulin 0 min (pmol/l)/p-glucose 30 min (mmol/l). Insulinogenic index of M132-2 declined from 50 to 33 and for M132-4 from 19 to 13. BMI for M132-2 increased from 19.7 to 24.8 kg/m^2^; for M132-4 BMI was unchanged, 24.2 kg/m^2^, over the 5-year period. The R6 residue is conserved across mammalian species (rhesus monkey, elephant, mouse, dog and horse) and xenopus but is not conserved in lizard (S), platus (G) or stikleback (Q). A mutation in the same codon, R6C, was recently described in an English MODY family affecting the proband, the mother and the maternal grandmother [3], underlining a putative importance of this residue for proper function of the signal peptide.

In a Czech MODY proband a previously described R46Q mutation was found (Figure 1B). The mutation is located at residue 22 of the B chain. The mutation co-segregates with diabetes in two affected relatives and is not present in any of the non-diabetic family members or 96 controls. The proband, a 17-year old girl, was diagnosed with diabetes at 13 years of age and had been treated with insulin since onset of disease. Her mother was diagnosed at 14 years of age with polyuria and polydipsia, and had been treated with insulin since onset of diabetes. The grandmother was diagnosed with diabetes at 35 years of age and treated with OHA and insulin. No coding mutations were found in GDM women or GAD-negative type 1 diabetic patients.

It is very likely that the R46Q and R6H mutations are disease causing and not just rare neutral polymorphisms. In already published studies [1,3,7,8] a total of 1560 individuals primarily of Northern European ancestry have been screened for *INS *mutations. The R46Q mutation was identified in one Norwegian MODY family [7]. The mutation segregated with diabetes in the family. The R6H mutation has not been identified in the 1560 examined individuals indicating that this mutation is not a rare polymorphism. Furthermore, we have screened additional 74 Danish individuals (data not shown) without identifying the R46Q and R6H mutations.

## Conclusion

Mutations in *INS *can be a rare cause of MODY and we conclude that screening for mutations in *INS *should be recommended in MODYX patients.

## Abbreviations

GAD: Glutamic acid decarboxylase; GDM: Gestational diabetes mellitus; INS: Insulin gene; MODY: Maturity-onset diabetes of the young; PNDM: Permanent neonatal diabetes mellitus.

## Competing interests

T.W Boesgaard, E.A Andersson, R Bergholdt, F Pociot, O Pedersen and T Hansen hold stock in Novo Nordisk, and O Pedersen and T Hansen have received lecture fees from pharmaceutical companies. All other authors declare that there is no competing interest associated with this manuscript.

## Authors' contributions

The concept and idea regarding the study belong to EAA, TWEB, OP and TH. The collection of study subjects was planned and performed by SP, OC, BO, JL, JL, PD, RB, FP, CP, TWEB, OP and TH.

The original hypothesis regarding the genetic study was conceived by EAA and TWEB, and approved by OP and TH. Detail planning of analyses and study design was performed by EAA and TWEB, and approved by FB, OP and TH. EAA, TWEB, OP and TH contributed to the establishment of study population databases specific for this study. Statistical analyses were performed by EA and TWEB. The first manuscript was written by TWEB and EAA (equal contributions) and the final draft was produced by TWEB, OP and TH. All authors revised the manuscript and contributed to the discussion.

## Pre-publication history

The pre-publication history for this paper can be accessed here:

http://www.biomedcentral.com/1471-2350/11/42/prepub

## References

[B1] Bonfanti R Colombo C Nocerino V Insulin gene mutations as cause of diabetes in children negative for five type 1 diabetes autoantibodies Diabetes care 2009 11 123 125 10.2337/dc08-0783 18840770 PMC2606844

[B2] Colombo C Porzio O Liu M Seven mutations in the human insulin gene linked to permanent neonatal/infancy-onset diabetes mellitus J Clin Invest 2008 11 2148 2156 18451997 10.1172/JCI33777 PMC2350430

[B3] Edghill EL Flanagan SE Patch AM Insulin mutation screening in 1,044 patients with diabetes: mutations in the INS gene are a common cause of neonatal diabetes but a rare cause of diabetes diagnosed in childhood or adulthood Diabetes 2008 11 1034 1042 10.2337/db07-1405 18162506 PMC7611804

[B4] Støy J Edghill EL Flanagan SE Insulin gene mutations as a cause of permanent neonatal diabetes Proceedings of the National Academy of Sciences 2007 11 15040 15044 10.1073/pnas.0707291104 PMC1986609 17855560

[B5] Murphy R Ellard S Hattersley AT Clinical implications of a molecular genetic classification of monogenic beta-cell diabetes Nat Clin Pract Endocrinol Metab 2008 11 200 213 10.1038/ncpendmet0778 18301398

[B6] McCarthy MI Hattersley AT Learning from molecular genetics: novel insights arising from the definition of genes for monogenic and type 2 diabetes Diabetes 2008 11 2889 2898 10.2337/db08-0343 18971436 PMC2570381

[B7] Molven A Ringdal M Nordbo AM Mutations in the Insulin Gene Can Cause MODY and Autoantibody-Negative Type 1 Diabetes Diabetes 2008 11 1131 1135 10.2337/db07-1467 18192540

[B8] Rubio-Cabezas O Edghill EL Argente J Hattersley AT Testing for monogenic diabetes among children and adolescents with antibody-negative clinically defined Type 1 diabetes Diabet Med 2009 11 1070 1074 10.1111/j.1464-5491.2009.02812.x 19900242

[B9] Jørgensen T Borch-Johnsen K Thomsen TF Ibsen H Glümer C Pisinger C A randomized non-pharmacological intervention study for prevention of ischaemic heart disease: baseline results Inter99 Eur J Cardiovasc Prev Rehabil 2003 11 377 386 10.1097/01.hjr.0000096541.30533.82 14663300

